# PP2A as the Main Node of Therapeutic Strategies and Resistance Reversal in Triple-Negative Breast Cancer

**DOI:** 10.3390/molecules22122277

**Published:** 2017-12-20

**Authors:** Henan Zhao, Duojiao Li, Baojing Zhang, Yan Qi, Yunpeng Diao, Yuhong Zhen, Xiaohong Shu

**Affiliations:** 1 Department of Pathophysiology, Dalian Medical University, Dalian 116044, China; 2 Kamp Pharmaceutical Co. Ltd., Changsha 410008, China; li_duojiao@126.com; 3 College of Pharmacy, Dalian Medical University, Dalian 116044, China; ares_dl@163.com (B.Z.); friendqy@163.com (Y.Q.); diaoyp@163.com (Y.D.); zhenyhwaner@aliyun.com (Y.Z.)

**Keywords:** breast cancer, TNBC, PP2A, resistance reversal, molecular targets

## Abstract

Triple negative breast cancer (TNBC), is defined as a type of tumor lacking the expression of estrogen receptor (ER), progesterone receptor (PR) and human epidermal growth factor receptor 2 (HER2). The ER, PR and HER2 are usually the molecular therapeutic targets for breast cancers, but they are ineffective for TNBC because of their negative expressions, so chemotherapy is currently the main treatment strategy in TNBC. However, drug resistance remains a major impediment to TNBC chemotherapeutic treatment. Recently, the protein phosphatase 2A (PP2A) has been found to regulate the phosphorylation of some substrates involved in the relevant target of TNBC, such as cell cycle control, DNA damage responses, epidermal growth factor receptor, immune modulation and cell death resistance, which may be the effective therapeutic strategies or influence drug sensitivity to TNBCs. Furthermore, PP2A has also been found that could induce ER re-expression in ER-negative breast cancer cells, and which suggests PP2A could promote the sensitivity of tamoxifen to TNBCs as a resistance reversal agent. In this review, we will summarize the potential therapeutic value of PP2A as the main node in developing targeting agents, disrupting resistance or restoring drug sensitivity in TNBC.

## 1. Introduction

Breast cancer is the most frequent cancer and the second leading cause of cancer-related mortality among women worldwide [1,2]. Triple-negative breast cancer (TNBC), which is defined by the lack of expression of estrogen receptor (ER), progesterone receptor (PR), and human epidermal growth factor receptor 2 (HER2), is the most fatal subtype of breast cancer and is associated with relatively poorer outcomes compared with other breast cancer subtypes [3,4,5,6]. Non-TNBC patients can be treated with specific therapies such as endocrine treatment in the event of ER and PR positivity (+), or the monoclonal antibody, trastuzumab, in the case of HER2(+) patients. As lacking hormone receptors and HER2, to date, no US Food and Drug Administration (FDA)-approved targeted therapies are available for TNBC. 

Recently, TNBC molecular subtypes were refined into four (TNBCtype-4) tumor-specific subtypes: basal-like 1 (BL1), basal-like 2 (BL2), mesenchymal (M), and luminal androgen receptor-like (LAR), to demonstrate differences in diagnosis age, grade, local and distant disease progression and histopathology [7,8]. Despite the rather aggressive clinical behavior of TNBC, however, BL1 had the highest pathological complete response (pCR) and BL2 and LAR the lowest [9]. The differences in clinical response and survival after neoadjuvant chemotherapy suggest that different subset of TNBC may be with vastly different biologies and responses to chemotherapy and targeted therapies [8,10,11,12,13,14]. However, with increased understanding of TNBC biology, a number of novel targets have emerged [15,16,17,18], which have great potential for drug sensitivity and therapeutic development for TNBC. 

Phosphoprotein phosphatase 2A (PP2A), a major serine/threonine phosphatase, appears to be critically involved in cellular growth control and potentially in the development of cancer through regulating a broad array of biological processes such as the cell cycle, DNA replication, transcription and translation, signal transduction, cell proliferation, cytoskeleton dynamics and cell mobility and apoptosis [19,20,21,22]. PP2A has also been found as a common alteration in breast cancer, and its alteration would lead to carcinoma aggressiveness and poor prognosis [23,24,25,26]. Furthermore, PP2A is widely involved in regulating most current developments in targeted therapeutic strategies for TNBCs [27,28], and more importantly, PP2A could induce ER re-expression in ER-negative breast cancer cells, and which would reverse endocrine therapeutic resistance in ER(−) breast cancer, then providing a potential therapeutic strategies for TNBC. Therefore, understanding the structure of PP2A and its interactions with associated signaling pathways can help us to shed light on its potential values as a novel strategy to enhance the efficacy of chemotherapy, as well as to overcome the drug resistances in TNBCs. 

## 2. Subunit Proteins of PP2A

**Figure 1:**
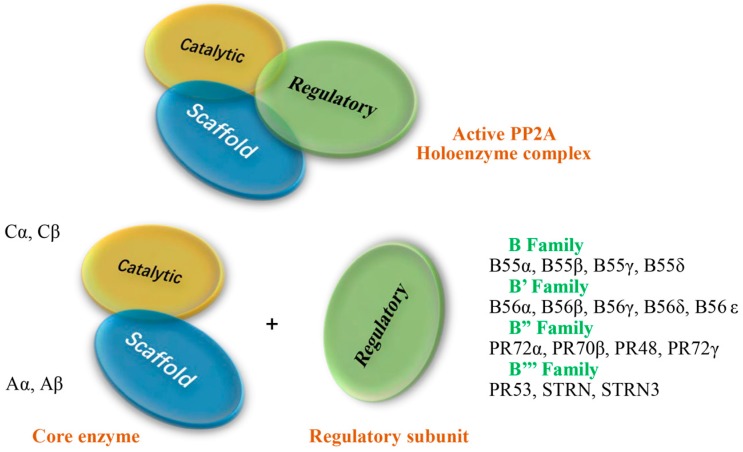
Diagram representing the PP2A Ser/Thr phosphatase complex. Regulatory subunits confer substrate specificity to the core enzyme.

Unlike many kinases (e.g., ERK, PKC, and AKT), PP2A is a multimer [29,30]. The predominant PP2A complex is a heterotrimer composed of an active core dimer that can exit independently, consisting of a catalytic subunit (C subunit ~36 kDa), a scaffold A subunit (65 kDa) and a variable regulatory B subunit [31,32,33,34]. There are two isoforms of the scaffold C subunit (PPP2CA aka Cα and PPP2CB aka Cβ), which are both ubiquitously expressed, and α isoform is about 10 times more abundant than the β isoform. The structural subunit A has two isoforms, PPP2R1A (Aα) and PPP2R1B (Aβ), which have high homology suggesting similar structure and function. The B subunit is categorized into four unrelated families: B (aka B55, gene symbol PPP2R2), B′ (aka B56, gene symbol PPP2R5), B″ (aka PR48/72/130, gene symbol PPP2R3) [31,32,33,34,35,36,37,38]. Striains (B‴ family; gene symbol STRN or PPP2R4) are a fourth regulatory subunit family (Figure 1) [34,38]. Although, the homologous sequence in protein A and C subunits are >80%, there are obvious differences between each isoform [32,33]. In fact, catalytic subunit of PP2A is responsible for the dephosphorylation events, and B regulatory subunit determines the substrate specificity and cellular localization of the resulting PP2A isoform, so PP2A isoforms are identified by the B regulatory subunit they contain.

## 3. PP2A Regulating Cell Cycle Control in TNBCs

The cell cycle covers a broad range of interconnected pathways but is more likely to be deregulated in TNBC. Cyclin E1, an activator of cyclin-dependent kinase 2 (CDK2), is an important regulator of the G1–S-phase transition of the cell division cycle. However, cyclin E1 is aberrantly overexpressed in many human cancers. Approximately 30% of breast cancers overexpress cyclin E1 [39,40]. Cyclin E1 overexpression is also a major molecular determinant of basal-like TNBC and an independent predictor of poor patient outcome [39,41]. Cyclin E1 overexpression has also been associated with increased resistance to trastuzumab in Her2+ breast cancers [42]. Tan and colleagues found that augmented PP2A-B55β expression stabilizes cyclin E1 and promotes its overexpression in cancer-derived cell lines and breast tumors, suggesting PP2A-B55b–directed therapies might be particularly effective for the treatment of highly aggressive basal-like TNBCs, whose growth and survival have been shown to depend on these abnormalities [39,43]. Like the B55 family, the B56 family also plays important role in cell cycle progression through separating chromosomes during mitosis by controlling the timing of kinetochore assembly and regulating spatial aspects of chromosome alignment with microtubules [44,45,46,47,48,49,50,51,52]. Xu et al. found that B56 subunits depletion resulted in impaired chromosome segregation due to misalignment of chromosomes and is independent of kinetochore assembly [49]. Although B55 and B56 can be functional competitors, sometimes, these two PP2A families also cooperate in mitotic regulation [44,45].

In triple-negative breast cancer, retinoblastoma protein (RB) and its regulatory cyclin-dependent kinases (CDK) have been reported as one of the key regulatory elements in the cell cycle progression. RB is phosphorylated by cyclin E/CDK2 in cooperation with cyclin D/CDK4/CDK6. RB pathway deregulation comprises ~20% of TNBC, and knockdown of retinoblastoma (RB1) in TNBC cell lines showed an increase of sensitivity to gamma-irradiation, doxorubicin and methotrexate treatments [15]. A recent study showed that DNA damage induced RB dephosphorylation in a PP2A-dependent manner, and this process could be inhibited by Pin1 [53]. Kolupaeva et al. demonstrated that p107 dephosphorylation was a key event in FGF-induced cell cycle arrest and indicated that in chondrocytes FGF activates the PP2A phosphatase to promote p107 of RB protein family dephosphorylation [54]. These studies suggest a novel molecular mechanism in which the PP2A-mediated modulation of RB phosphorylation has an important role in cancer treatment.

## 4. Relevance of PP2A Controlling DNA Damage Responses and PARP Inhibitors in Breast Cancer

Poly(ADP-ribose) polymerase (PARP) inhibitors are currently emerging as one of the most promising targeted therapeutics to treat TNBCs. Targeting the DNA repair complex of PARP inhibitor is a novel, biological approach currently being evaluated. PARP-1 is an important enzyme involved in the repair of single-strand DNA breaks as a part of the base excision repair pathway. In BRCA deficient cancer cells, which are deficient in DNA damage-sensing and the homologous recombination (HR) dependent DNA-repair pathway [55], inhibition of PARP activity will lead to an increase in double-strand DNA breaks. Therefore, *BRCA-1* and *BRCA-2* defective cells are markedly sensitive to PARP inhibitor. The incidence of *BRCA1* mutation carriers in TNBC patients is very high at about 70% and indicates a particularly poor prognosis in these patients [15,56]. The DNA damaging agents are represented by a large class of drugs used in TNBC called antineoplastic drugs including alkylating agents, anthracyclines antibiotics, antimetabolites and platinum salts. 

In 2014, the first drug targeting DNA repair defects, the PARP inhibitor Lynparza^®^ (olaparib, KuDOS Pharmaceuticals/Astra Zeneca), was approved by the FDA as the first ‘personalized therapy’ for advanced *BRCA1/2* mutated ovarian cancer [57,58]. In TNBC patients, cytoplasmic PARP expression predicts high sensitivity to anthracycline-taxane based chemotherapy [59]. In fact, PARP inhibitors are currently emerging as one of the most promising targeted therapeutics to treat TNBCs [15].

The role of PP2A in the tumor cell survival is understudied and some work has been done on PP2A role in DNA damage responses. Reports on the PR72/PR130 subunits show that these PP2A isoforms support pro-survival signaling and metastasis [60,61]. At least for these B subunit family members, there could be clinical benefit to inhibiting their function. Wei and colleagues demonstrated that LB100, a non-toxic PP2A inhibitor, sensitized pancreatic cell lines to radiation via mechanisms involving disruption of DNA repair [62]. Kalev et al. reported that suppression of 4 different PP2A regulatory B subunits (PPP2R2A, PPP2R2D, PPP2R5A, and PPP2R3C) impaired the efficiency of DNA repair, suggesting that these specific PP2A complexes were involved in control of DNA repair pathways. The Sablina Laboratory has demonstrated that low expressions of B55 alpha in lung cancer cells was associated with increased phosphorylation of ATM and reduced levels of *BRCA1* and *RAD51* [63]. Cells with reduced B55 alpha were sensitive to PARP inhibitors because these cells were less efficient at DNA repair. These examples suggest that targeting PP2A can have therapeutic benefits in TNBC treatment. 

## 5. Epidermal Growth Factor Receptor and PP2A Regulation

Epidermal growth factor receptor (EGFR) is a receptor tyrosine kinase (RTK) that belongs to the ErbB family, and a transmembrane protein comprising an extracellular ligand binding domain, transmembrane domain, and cytoplasmic tyrosine kinase domain [64,65,66,67]. The EGFR gene is frequently mutated or overexpressed in lung, colon, head and neck, brain, pancreatic, and breast cancers, and which promotes tumor progression and drug resistance in these cancers [68,69,70,71]. Overexpression of EGFR in cancer is partly due to gene amplification [64,72], but the underlying mechanisms are not yet fully elucidated [64].

TNBCs have the highest rate of EGFR overexpression with a frequency ranging from 13% to 78% [73,74], depending on the ethnicity and the detection methods [64]. Moreover, EGFR expression has been identified as a valuable independent prognostic marker to predict clinical outcomes [75,76,77]. In a phase II trial cetuximab (an anti-EGFR antibody) in combination with carboplatin showed a response rate of 20% [78]. Therefore, EGFR is considered an attractive therapeutic target for EGFR inhibitors in TNBC.

In fact, suppression of PP2A appeared to be associated with ErbB2-mediated carcinogenesis [24,79]. Wong et al. found that inhibition of the PP2A catalytic subunit could induce apoptosis through p38 MAPK, Caspase 3, and PARP activation in ErbB2 overexpressing breast cancer cells [80]. Janssens’ laboratory identified the PR130/B”α1 (PR130) regulatory B-type subunit of PP2A as an interaction partner of SHIP2, and demonstrated how knockdown of PR130 affects EGFR degradation and EGF-mediated signaling [49]. These studies suggest that PP2A subunits could be another way to overcome resistance to EGFR therapy in TNBC.

## 6. Benefits of PP2A Activity in Immunotherapy for Breast Cancer Patients

Rapid progression and the development of resistance are the main challenges of the TNBC treatment. Several large retrospective analyses from individual clinical trials have demonstrated that tumor infiltrating lymphocytes (TILs) are prognostic in early-stage TNBC [7,81,82,83,84]. Meanwhile, gene expression profiling revealed an association between expression of immunomodulatory genes and better clinical outcomes in TNBC. As the understanding of the complex interplay between breast cancer biology and immunity is expanding, it may offer new advances in immunotherapy for breast cancer patients.

Programmed cell death protein 1 (PD-1) is an inhibitory co-receptor expressed on activated and exhausted T cells and B cells, monocytes and natural killer T cells. Further, cytotoxic T-lymphocyte antigen 4 (CTLA-4) and PD-1 are 2 key cell-surface receptors and suppress AKT mediated signaling upon ligation with their associated ligand (PL-L1 or PD-L2 for PD-1, CD28 for CTLA4) [29]. In the PD-1 pathway, PD-L1 can directly lead to death of reactive T cells. Further, primarily in TNBC [85,86], PD-L1 is associated with the presence of tumor infiltrating lymphocytes (TILs) [87] and correlates with higher histological grade [88]. Therefore, current therapeutic strategies mostly engage in blocking these immune checkpoints such as CTLA-4, PD-1 and PD-L1 [16]. Riley group provided direct evidence that PP2A played a prominent role in mediating CTLA-4 suppression of T-cell activation through PI3K/AKT pathways [89]. The Madrenas group further demonstrated that PP2A interacted with the cytoplasmic tail of human CTLA-4 through two motifs, the lysine-rich motif centered at lysine 155 and the tyrosine residue 182. This interaction and the phosphatase activity of PP2A were important for CTLA-4-mediated T cell activation [90]. In addition, they reported that except the catalytic subunit of PP2A, the regulatory subunit of PP2A also interacted with the cytoplasmic tail of CTLA-4 [91]. The association between PP2A and CTLA-4 involves a conserved three-lysine motif in the juxtamembrane portion of the cytoplasmic tail of CTLA-4. Mutations of these lysine residues prevent the binding of PP2A and enhance the inhibition of *IL-2* gene transcription by CTLA-4, indicating that PP2A represses CTLA-4 function [91].

Additionally, Villagra found that histone deacetylases (HDACs) down-regulated the expression of PD-L1 which activated the inhibitory regulation pathway PD-1 in T-cells [92]. This novel mechanism of PD-L1 regulation was mainly mediated by the influence of HDAC6 over the recruitment and activation of STAT3. Their data clearly demonstrated that a possible explanation for the impaired phosphorylation of STAT3 in the absence of HDAC6 could be originated by the enhanced interaction of PP2A with STAT3, which in turn could facilitate the dephosphorylation of STAT3-mediated by PP2A [92].

These findings reveal the requirement for proper balance of PP2A activity in immune modulation through multiple regulatory mechanisms, including CTLA-4 and PD-1, and provide a key pre-clinical rationale and justification to further study PP2A regulators as potential immuno-modulatory agents in TNBCs.

## 7. Effect of PP2A on the Cell Death Resistance Mechanisms

### 7.1. PP2A, an Undeniable Regulator of Apoptosis Escape

Inducing apoptosis is the major aim of chemotherapeutic treatments in TNBC, as apoptosis escape is one of the most common causes of therapeutic resistance [15]. Gene mutation in apoptosis pathways has frequently been observed in TNBC and has been proved to be correlated with chemotherapy [93]. Using human breast cancer cell lines, Li et al. found that CIP2A (cancerous inhibitor of PP2A) depletion significantly induced caspase-3 activation, followed by anti-PARP cleavage in two TNBC cell lines, suggesting that PP2A can induce caspase-dependent apoptosis in TNBC cells [26]. In fact, the B56 family (PPP2R5 series) is critical for its regulation of molecules involved in apoptotic control including Bcl2, p53, MYC, Glycogen Synthase Kinase 3 (GSK3), the Extracellular Receptor Kinases (ERKs) and beta catenin [29,94,95,96,97,98,99,100,101,102,103]. B56 alpha has been shown to dephosphorylate Bcl2 at Ser 70 in response to stresses associated with ceramide production [29,94,96,104,105].

Bcl2 is an important protein in regulating apoptosis. Although its role in therapeutic resistance in TNBC was still uncertain, low Bcl2 increased the risk of death and recurrence, and Bcl2 expression seemed to predict the outcome of treatment [106,107]. It has been shown that the potent apoptotic agent ceramide (a potent apoptotic agent) activates a mitochondrial PP2A and promotes dephosphorylation of the anti-apoptotic molecule Bcl2 [96]. In that study, dephosphorylation of Bcl2 appears to be required for ceramide-induced cell death, because treatment of cells with low doses of the PP2A inhibitor, okadaic acid (OKA), could block Bcl2 dephosphorylation and promote cell survival [96]. Another study from the Ruvolo group revealed that overexpression of B56α promoted mitochondrial PP2A activity and Bcl2 dephosphorylation and potentiates cell killing with ceramide [96]. These dephosphorylation events were thought to occur within the flexible loop domain (FLD) of Bcl2, including the key S70 residue, known to be important for Bcl2’s anti-apoptotic function [108,109]. More specifically, phosphorylation occurred within the FLD of Bcl2 increased its pro-survival activity by increasing its association with Bax and decreasing its association with p53 [110,111]. Therefore, PP2A-B56α-mediated dephosphorylation of Bcl2 decreased its prosurvival activity, supporting a tumor suppressor role for PP2A-B56α. 

Another approach for triggering apoptosis is targeting TNF-related apoptosis-inducing ligand (TRAIL). Normally, TRAIL binds death receptors 4 and 5 then triggers the death-inducing signaling complex (DISC) which phosphorylates caspase-8 and initiates the extrinsic apoptosis pathway. Many cancers including breast cancer, exhibit disruption of TRAIL-mediated signaling, contributing to chemotherapeutic resistance and tumor metastasis [112,113,114,115]. An in vitro study demonstrated that TNBC with mesenchymal features may more benefit from TRAIL targeted therapy than epithelial phenotype TNBC [116]. PP2A has been shown to dephosphorylate and therefore inhibit Src, a non-receptor tyrosine kinase that has been shown to regulate TRAIL resistance in breast cancer cells through mediation of Akt-pathway survival signaling [117,118]. Xu et al. found that TRAIL treatment activated Src at tyrosine 418 through PP2A/C degradation, and in turn phosphorylated caspase-8 phosphorylation, then initiated apoptosis [119]. As interest for therapeutic development of TRAIL agonists continues to rise, PP2A inhibitor may guarantee further study as an adjuvant therapy, particularly in TRAIL-resistant cancers.

### 7.2. Role of PP2A in Autophagy Ambiguity

Autophagy, type II programmed cell death, is an evolutionarily conserved, self-degenerative process has been associated with both cell viability and death. It is often expressed at a basal level in cells representing its role in the recycling of proteins and tissue homeostasis [93]. However, in cancer, the overall role of autophagy is far more controversial with its inhibition and induction both showing beneficial and negative effects of tumor cell survival [93,120,121,122]. The role of autophagy in TNBC’s therapeutic approach has not been fully explored, but various elements from in vivo and vitro studies have been added to complete the complex puzzle that draws up the interplay between PP2A and autophagy pathway, suggesting PP2A to regulate autophagy and cell-survival.

A recent study reported that B55 alpha was involved in negative regulation of autophagy by serving as a Beclin 1 phosphatase [123]. Fujiwara and colleagues reported that B55 alpha is involved in negative regulation of autophagy by serving as a Beclin phosphatase [123]. They identified Ser-90 in Beclin 1 as a regulatory site whose phosphorylation was markedly enhanced in cells treated with okadaic acid, an inhibitor of PP2A. Interestingly, it appears that this mechanism is cell type specific as the phenomenon was observed in cells from skeletal tissue but not from the liver. Starvation resulted in B55 alpha dissociation form Beclin 1 allowing for Death-associated Protein Kinase 3 (DAPK3) phosphorylation of the autophagy molecule [29,123]. However, Wong et al. found that starvation triggers the release of PP2A from this latent complex containing Alpha4, resulting in rapid dephosphorylation of ULK1 and autophagy induction. ULK1 is subject to mTORC1 regulation, so this suggests an interesting cross-talk between AKT/mTOR components in autophagy [124]. Therefore, The B55 alpha subunit clearly has diverse roles in sustaining cellular homeostasis.

Additionally, p62/SQSTM1 has an important function in promoting survival signals. In TNBC, p62 was found overexpressed and correlated with advanced stage, higher proportion of lymph nodes and lymphovascular invasion, as well as higher risk of distant metastasis [125]. p62 can be further degraded along with ubiquitinated proteins in autolysosomes [126]. Consequently, as a result of the suppression of autophagy, blockade of PP2A is expected to induce an accumulation of ubiquitinated protein aggregates positive for p62. Magnaudeix et al. revealed that blockade of PP2A activity, either by OKA or by PP2Ac silencing, induced substantial relocalization of p62 from the cytosol and an overall increase in protein ubiquitinylation [127]. These data confirmed that p62 aggregates when PP2A was inhibited.

## 8. Significance of PP2A as Regulator of Estrogen Receptor

TNBCs are characterized by the lack of expression of ER, PR, and HER2. Absence of the estrogen receptor alpha (ERα) in human breast cancer cells is an indicator of poor prognosis, and predictive of lack of response to hormonal therapy. In fact, PP2A has been reported to regulate ER expression through different contributing molecular mechanisms, including not only transcriptional activation at the gene promoter but rather an increased ER mRNA stability and half-life [30,127]. In that study, inhibition of PP2A using the pharmacologic inhibitor okadaic acid or specific PP2A silencing, all reduced ER expression [127]. Liu reported that PP2A could dephosphorylate ER, thereby downregulating of ERα-estrogen response element (ERE) binding and inhibiting ERE-mediated transactivation of certain target genes such as *PR* [128]. In additionally, PP2A inhibition has been found markedly associated with negative ER and PR expression in breast cancer patients [128], and further determines doxorubicin resistance in breast cancer cells, which are re-sensitized after pharmacological restoration of PP2A activation using FTY720, an FDA-approved immune-suppressant drug [129]. FTY720 displays its anticancer properties through inducing PP2A dephosphorylation. The mechanism involves targeting both SET and CIP2A, thereby leading to PP2A activation [129,130]. Most importantly, FTY720 can induce ER re-expression in ER-negative breast cancer cells. Additionally, this ER increase after being treated with FTY720 has also been attributed to its inhibitory action of class I histone deacetylases (HDACs), which has been recently reported to affect PP2A activation by initiating CIP2A transcription [131]. Therefore, the potential therapeutic value of FTY720 to induce ER re-expression in TNBCs should be further investigated.

## 9. PP2A-Activity Regulating Agents/Drugs

Several previous studies have demonstrated that CIP2A and SET (two main endogenous PP2A inhibitors) play a role in regulating drug sensitivity in TNBCs [132,133,134,135]. Cancerous Inhibitor of PP2A (CIP2A) is a PP2A-interacting protein. Encoded by the *KIAA1524* gene, CIP2A is overexpressed and may be prognostic in lung cancer, breast cancer, and ovarian cancer [136,137,138]. It is most strongly associated with inhibiting the activity of PP2A on *c-MYC* resulting in *c-MYC* stabilization and consequential proliferation. Up to now, CIP2A has been targeted using natural compounds, erlotinib derivatives or small molecules, such as celastrol, ethoxysanguinarine, bortezomib and lapatinib [139,140]. SET, also known as inhibitor-2 of PP2A (I2PP2A), binds to the C subunit of PP2A. In addition to its overexpression, altered phosphorylation of SET also inactivates PP2A [141,142]. One strategy to inhibit SET involves ApoE, which binds to SET resulting in activation of PP2A [132,143,144]. In fact, activation of PP2A also represents a promising strategy for therapeutic intervention. FTY720 and its chiral deoxy analog drugs were used to indirectly reactivate PP2A by partly blocking the PP2A inhibitor protein SET [145]. Moreover, there have been orally bioavailable small molecule activators of PP2A (analogues of tricyclic neuroleptics that have been reported to activate PP2A through direct binding of the PP2A Aα subunit) [146]. Considering the PP2A structure, function and interaction with molecules involved in therapeutic strategies and drug resistance, targeting of these PP2A-activity regulating agents/drugs could also be a promising approach in TNBC’s treatment.

## 10. PP2A as a Main Node in Treatment in TNBC Subtype

TNBC is a diverse entity for which additional subclassifications beyond basal and non-basal may be needed. Recently, Lehmann et al. classified TNBC into four molecular subtypes: BL1, BL2, M and LAR based on gene expression profiles [7,8]. The differences of TNBCtype-4 in clinical response and survival after neoadjuvant chemotherapy suggest that different subset of TNBC may be with vastly different biologies and responses to chemotherapy and targeted therapies. The BL1 subtype is characterized by elevated cell cycle and DNA damage response gene expression, while the BL2 subtype is enriched in growth factor signaling [8]. M tumors are composed of genes encoding immune antigens and core immune signal transduction pathways [8]. The LAR subtype of TNBC is characterized by expression of the androgen receptor (AR) in the presence of a luminal gene expression and, might be treated with agents that target AR, as is the case of prostate cancer [12]. Of these, PP2A subunits play an important role in controlling of cell cycle (B55 and B56 families), DNA damage responses and regulating of tumor cell survival (B, B′ and B′′ subunits of PP2A). In addition, the catalytic and B56 subunits were reported to be able to affect EGFR degradation and EGF-mediated signaling. The activation of PP2A can also play prominent roles in CTLA-4 and PD-1-mediated immune modulation. While the LAR subtype of TNBC showed was shown to depend on AR signaling, and high response rate to anti-androgens in preclinical studies and clinical trials [147,148,149]. In fact, an increasing number of evidences support a crucial role for PP2A inhibition in AR signaling reactivation [150,151,152]. Moreover, it has been recently reported that inhibition of the PP2A/SET axis using OP449 (a SET antagonist peptide) led to overcome enzalutamide resistance in prostate cancer [27]. These evidences significantly suggest that targeting different subunit of PP2A appears to be a promising treatment for different subtype TNBCs, e.g., targeting DNA-repair deficiency by B, B′ and B′′ subunits of PP2A are more likely to be effective for BL1-TNBC.

## 11. Conclusions

**Figure 2:**
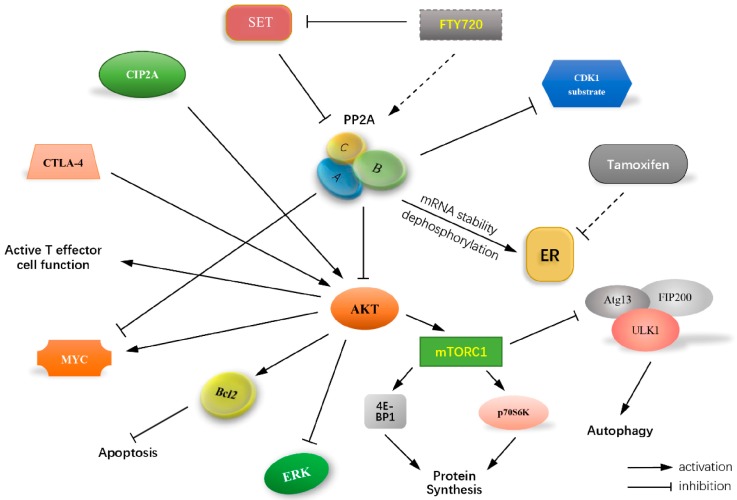
Role of PP2A regulating potential therapeutic targets for TNBCs. PP2A is widely involved in regulating most current developments in targeted therapeutic strategies for TNBCs, including cell cycle, DNA damage, EGFR degradation, immune responses, cell death resistance and ER or AR regulation. A better understanding of the structure of PP2A and its interactions with associated signaling pathways can give us a great appreciation of the crucial roles these phosphatases play in adapted therapeutic approaches for each type of TNBC and even resistance reversal as an importantly main node.

Establishment of an effective therapy for TNBCs due to the restricted therapeutic strategies relative to the lack of expression of ER/PR/HER2 and intrinsic or developed resistance still remains as a great challenge in breast cancer therapy. As mentioned in this review, one of the most important concepts in targeted TNBC chemotherapy is consideration of overlapping molecular tumor-specific alterations induced by cytokines, ROS, and hypoxia. A better understanding of PP2A regulation in cell cycle, DNA damage, EGFR degradation, immune responses, cell death resistance and ER or AR regulation (Figure 2) gives us a great appreciation of the crucial roles these phosphatases play in adapted therapeutic approaches for each type of TNBC and even resistance reversal as an importantly main node.
